# Deciphering lncRNA and circRNA control of autophagy in osteosarcoma mechanisms and clinical translation

**DOI:** 10.1007/s12672-026-04629-6

**Published:** 2026-03-20

**Authors:** Sayed Amir Mohammad Hosseini, Behnaz Mahmoodieh, Mehrdad Hashemi, Elnaz Asadifard, Fatemeh Hassanzadeh, Zahra Hassanzadeh, Bita Fazel, Neda Hedayati, Kiavash Hushmandi, Seyed Ali Olianasab, Alireza Mafi, Seyedeh Mahdieh Khoshnazar, Mina Alimohammadi, Najma Farahani, Ehsan Maghrebi-Ghojogh, Afshin Taheriazam, Amirhossein Zabolian

**Affiliations:** 1 Department of Clinical Sciences, Faculty of Veterinary Medicine, Shahid Chamran University of Ahvaz, Ahvaz, Iran; 2 Young Researchers and Elite Club, Tehran Medical Science, Islamic Azad University, Tehran, Iran; 3 Farhikhtegan Medical Convergence Sciences Research Center, Farhikhtegan Hospital, Faculty of Medicine, TeMs.C., Islamic Azad University, Tehran, Iran; 4 Department of Genetics, Faculty of Advanced Sciences and Technology, TeMs.C., Islamic Azad University, Tehran, Iran; 5 Medical Genomics Research Center, TeMs.C., Islamic Azad University, Tehran, Iran; 6 Department of Laboratory Sciences, School of Paramedical Sciences, Shiraz University of Medical Sciences, Shiraz, Iran; 7 Faculty of Veterinary Medicine, University of Tehran, Tehran, Iran; 8 School of Medicine, Iran University of Medical Science, Tehran, Iran; 9 Nephrology and Urology Research Center, Clinical Sciences Institute, Baqiyatallah University of Medical Sciences, Tehran, Iran; 10 Department of Plant Science, Faculty of Biological Sciences, Kharazmi University, Tehran, Iran; 11 Department of Genetics and Biochemistry, School of Medicine, Arak University of Medical Sciences, Arak, Iran; 12 Physiology Research Center, Institute of Neuropharmacology, Kerman University of Medical Sciences, Kerman, Iran; 13 Department of Immunology, School of Medicine, Shahid Beheshti University of Medical Sciences, Tehran, Iran; 14 Pharmaceutical Sciences Research Center, Faculty of Pharmacy, Mazandaran University of Medical Sciences, Sari, Iran; 15 Department of Orthopedics, Faculty of Medicine, TeMs. C., Islamic Azad University, Tehran, Iran; 16 Department of Orthopedics, Shahid Beheshti University of Medical Sciences, Tehran, Iran

**Keywords:** Noncoding RNA, CircRNA, LncRNA, Autophagy, Osteosarcoma, Signaling pathway

## Abstract

Osteosarcoma (OS) is the most common aggressive bone cancer, which predominantly affects children and adolescents. It has high metastatic potential and poor survival in advanced stages. Despite the advancements in multimodal therapy, drug resistance and recurrence remain daunting. Autophagy plays a dual role in OS development-suppressing early tumorigenesis but facilitating the survival of tumor cells under stress. These include major regulators such as non-coding RNAs (ncRNAs), specifically long non-coding RNAs (lncRNAs) and circular RNAs (circRNAs). These ncRNAs regulate autophagy through binding to microRNAs and modulating related signaling pathways. Their dysregulation contributes to OS cell proliferation, metastasis, immune evasion, and chemoresistance. This review summarizes current knowledge on the regulation of autophagy by lncRNAs and circRNAs in OS development. Some are oncogenic drivers that induce autophagy and drug resistance, and others are tumor suppressors that inhibit autophagy and increase drug sensitivity. Some regulatory axes, lncRNA MEG3/miR-21-5p/p53, OIP5-AS1/miR-153/ATG5, Sox2OT-V7/miR-142/miR-22, and circMRPS35/FOXO3, illustrate the complex manner in which ncRNAs affect autophagy and treatment response. Clarification of these networks enlightens OS biology and indicates ncRNAs as potential diagnostic, prognostic, and therapeutic targets.

## Introduction

Osteosarcoma (OS) is the most common primary malignant bone tumor of mesenchymal origin, with heterogeneity, high pulmonary metastatic propensity, and chemosensitivity [1, 2]. It accounts for approximately 2–3% of all childhood cancers and up to 20% of primary bone tumors in the world [2, 3]. OS is most frequently seen in the metaphyseal segments of long bones, most often in the distal femur and proximal tibia, and in children, adolescents, and, less frequently, adults [4, 5]. Despite advances in multimodal treatment regimens including neoadjuvant chemotherapy, surgical excision, and adjuvant chemotherapy, recurrence and chemoresistance remain the primary causes of poor prognosis, decreasing survival to around 20% in metastatic to 67% in localized disease [6–8].

The pathogenesis of OS is marked by the malignant transformation of mesenchymal cells through osteoblastic differentiation [9]. Genetic studies showed mutations in key tumor suppressor genes such as TP53 and RB1, and abnormalities in DNA repair and signaling pathways, resulting in genomic instability and heterogeneity of tumors [10–12]. Some of these genes also control autophagy, linking genetic abnormality to cellular stress responses [13].

Autophagy is an evolutionarily conserved process of cellular degradation and recycling, serving to maintain homeostasis, remove damaged organelles, and ensure survival under stress [14]. Autophagy is involved in osteoblast differentiation, osteoclast function, and bone matrix mineralization in bone biology [14, 15]. Dysregulation of autophagy has been involved in several bone disorders, including OS, osteoporosis, and Paget’s disease [16]. Aberrant expression of autophagy regulators such as TP53, RB1, IGF2, H19, COPS3, and RUNX2 shows that impaired autophagic regulation is involved in the onset and progression of OS [17, 18].

Non-coding RNAs (ncRNAs) have been recognized as critical autophagy regulators [19]. Although they encode only about 2% of the human transcriptome for protein production, the remaining 98% are ncRNAs with a variety of regulatory functions [20]. MicroRNAs (miRNAs), long non-coding RNAs (lncRNAs), and circular RNAs (circRNAs) could regulate autophagy at the transcriptional and post-transcriptional levels by downregulating autophagy-related genes [21, 22]. The first ncRNA discovered as an autophagy regulator was miR-30a [23], and subsequent research identified that lncRNAs and circRNAs are capable of both inducing or inhibiting autophagy, and hence, tumorigenesis, metastasis, and chemoresistance are affected [24–27].

Recent evidence highlights lncRNAs and circRNAs as both important autophagy modulators in OS development. This review synthesized the current evidence of how lncRNAs and circRNAs regulate autophagy and their potential diagnostic and therapeutic significance in OS.

## Osteosarcoma: genetic and molecular landscape

After chordoma and chondrosarcoma, OS is the third most common primary bone malignancy in adults, but it is the most common in pediatric and adolescent groups. The global incidence is about 3.4 cases per million annually [28]. In the United States, approximately 900 new cases of OS are reported yearly. OS affects mostly children and adolescents in the age range between 10 and 30. More precisely, the distribution of OS is bimodal with a peak between 15 and 19 years old (8 cases/million/year) and another peak between 75 and 79 years old (6 cases/million/year) [29]. The first peak of the teenage group is due to intense linear bone development. Long bones are also common sites for tumors, more so in regions of rapid growth, which include arms, legs, knees, and shoulders. The increased incidence of these cancers in young individuals may be influenced by possible genetic predispositions to cancer risk syndromes [30]. Risk factors in the pathophysiology of OS include disruptions in the signaling pathways of TP53, Rb, RecQLike helicase 4, Bloom Syndrome RecQLike helicase, and Werner Syndrome RecQ helicase. Thus, OS is more likely to develop in children and adolescents with genetic syndromes such as Li-Fraumeni, hereditary retinoblastoma, Rothmund-Thomson, Bloom, or Werner syndrome [31]. Increased bone resorption by osteoclasts, coupled with an increased incidence of Paget’s disease of bone (PDB), accounts for the second peak in OS in the elderly. In addition, OS in older adults is also influenced by lifetime exposures to environmental substances such as radium, beryllium, and chromium, along with a history of radiation exposure, like prior incidents of radiation therapy [32, 33].

An unusual geographic pattern characterizes the incidence of OS. Both Asian (Chinese, Japanese, and Indian) and some Latin American groups have low OS prevalence [34]. With a male-to-female ratio of 1.28:1 in the 25–59 age group and an even higher ratio of 1.43:1 in the 0–24 age group, OS is more common in men than in women in the majority of countries. Moreover, this ratio differs by population. For instance, in Western Europe, the incidence of OS is higher in females aged 60 + years compared to males of the same age, while in Australia and Canada, the incidence is even higher in males aged 75+ [28]. OS is a primary malignant tumor of the skeleton that primarily affects the long bones, whereby osteoid tissue or immature bone is formed by sarcoma cells. Further sub-classification of the tumors according to their distinctive features can be done based on the most common type of stromal differentiation, which includes osteoblastic, fibroblastic, chondroblastic, small-cell, telangiectatic high-grade surface, or extraskeletal types. Histologically, the three groups that can be identified include low-grade, including the subtypes of periosteal and parosteal; intermediate-grade; and high-grade, which includes most of the subtypes [35]. The most common type, so-called “conventional OS,” is a well-produced osteoid-producing tumor arising intramedullary; it comprises 85% of all OS cases in children and adolescents [33].

The genomic architecture of OS tumors is generally characterized by extensive structural changes, suggesting the action of multiple mutational mechanisms, including chromothripsis, chromoplexy, kataegis, and other structure-altering processes [36]. Concurrently, there are recurrent amplifications of MYC, EGFA, and CCNE1 along with genome-wide copy-number changes, often characterized by a prevalent loss of copy number, with PTEN and CDKN2A/B being the most frequently affected [37]. Whole-genome duplication is common in OS tumors, likely due to the challenges that the extensive copy number losses present [38]. Traditionally viewed as a hallmark of chromosomal instability, this genomic complexity may, as recent reports have suggested, actually arise from mechanisms inducing complexity at the earliest stages of malignant transformation. Notably, even from diagnosis to relapse, the complex genomes generated are subsequently maintained with some degree of fidelity [8].

Aside from these characteristic structural changes, only modest levels of point mutations and a low number of commonly altered genes have been reported by large-scale sequencing [39]. The most frequently mutated gene is the tumor suppressor TP53, which is lost from more than 90% of OS tumors. The majority of these loss events are caused by deletions or rearrangements in intron 1, rather than point mutations [40]. The main function of this gene is to induce apoptosis in cells containing mutations. The loss of this guardian function is crucial for the survival of cancer cells, especially since OS is mostly marked by a chaotic genome. Up to 30% of OS tumors also contain RB1 deletion, often due to loss of heterozygosity (LOH) [8]. The initial phases of the transformation process most likely involve the inactivation of TP53 and RB1 (inactivation of TP53 is a prerequisite for the proliferation of abnormal genomes), followed by the rapid accumulation of driver alterations like amplification of MYC, loss of PTEN, and deletion of ATRX. This deletion likely leads to alternative lengthening of telomeres and is linked with poor survival [41]. Such genomic alterations can be used as pointers to trace tumor evolution. Many tumor suppressors commonly inactivated in OS were demonstrated to play a role in the regulation of autophagy [42]. It was shown that RB1, ARF, WIF1, PTEN, and TSSC3 all cause autophagy by distinct mechanisms [43]. More recently, it was also shown that RECQL4 controls autophagy through two distinct mechanisms [44]. Finally, some studies showed that OS has an overactivated mTOR pathway, indicative of autophagy suppression [45]. Taken together, these findings reveal that OS impacts a high number of autophagy regulators, pointing to the fact that this important process is dysregulated [17].

## Autophagy: mechanisms and signaling in cancer

Autophagy is a cellular self-degradation and intracellular recycling mechanism that has been conserved during evolution and maintains metabolic and homeostasis stability. Autophagy is induced by various cellular stressors, including nutritional deprivation, organelle damage, and misfolded protein accumulation [46]. This autophagic mechanism has been implicated in both cell survival and death [47]. During nutritional deprivation, autophagy is enhanced to make vital proteins and other nutrients available for use as an energy source, thus promoting cell survival [48]. Recent studies have demonstrated that hypoxia may regulate autophagy and initiate mechanisms that reduce the oxidative stress caused by low oxygen levels [49].

**Fig. 1:**
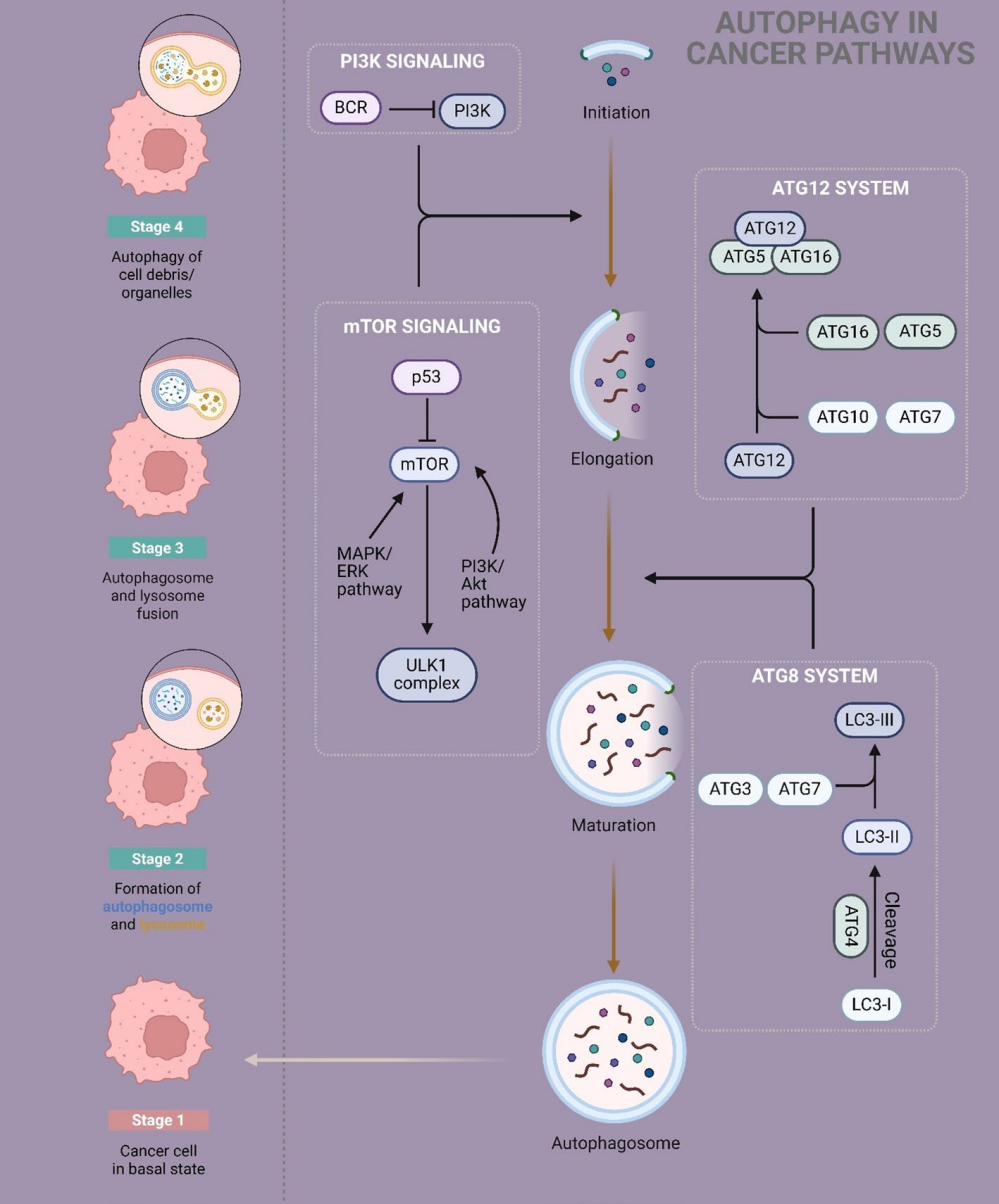
An overview of autophagy processes and their dual involvement in cancer development

The basal levels of autophagy are utilized by the cell in normal conditions to help biological function, homeostasis, quality control of cell contents, and removal of organelles and outdated proteins [50]. Moreover, the maintenance of distinctive properties of stem cells, namely, self-renewal and differentiation, is associated with autophagy [51]. As demonstrated in Fig. 1, autophagy has a dual role in cancer by balancing tumor suppression and survival. At some levels, autophagy supports the proliferation of cancer cells and tumor growth, while it can also suppress carcinogenesis and prevent cancer cells’ survival, leading to cell death [52].

Several proteins regulate the mechanism of the autophagic process. Mammalian target of rapamycin (mTOR) is associated with stress, cancer development, and cell division. The two complexes of mTOR, mTORC1 and mTORC2, have different roles and locations [53]. Activated mTORC1 inhibits autophagy and is required for the phosphorylation of autophagy-related protein (ATG). Autophagy is induced when mTORC1 is inhibited in response to a range of stressful conditions, such as starvation and organelle damage. AMP-activated protein kinase (AMPK) regulates mTORC1, and the induction of autophagy is stimulated by high AMPK and inhibition of mTORC1. However, it is still not known how mTORC1 acts during autophagy [54].

When mTORC1 is inhibited, the Unc-51-like autophagy-activating kinase (ULK) complex is dephosphorylated and activated [55]. When the activated ULK complex binds to the phagophore, class III PI3K is activated. Then, beclin-1 recruits many proteins involved in the maturation and elongation of the autophagosomes [56]. Autolysosomes are formed when mature autophagosomes merge with lysosomes and, through a process of autophagy, degrade dysfunctional organelles and proteins [54].

Compared to apoptosis, less information is available on autophagy genes. Mutations in the ATG2, ATG5, ATG9, ATG12, and UVRAG genes have been linked to microsatellite instability in malignancies. Some tumors have deletions in the Atg6 (Beclin1) gene, whereas human malignancies infrequently have point mutations [57]. The role of autophagy in cancer is a matter of debate. Whereas some data support the hypothesis that autophagy suppresses tumor growth, other data suggest that autophagy promotes tumor growth and protects tumors against cell death [58]. A cell death process involving both autophagy and apoptosis is selectively inactivated when primary epithelial cells acquire immortality, and contributes to the luminal structures in the development of epithelial model systems of mammary acini [59]. This would indicate that autophagy represses the formation of an epithelial tumor at an earlier stage. Taken together, these findings would suggest that either inducing or repressing caAs a result of the marked elevation in autophagy during starvation, the cell acquires the ability to degrade proteins and organelles and access macromolecular precursors, including amino acids, fatty acids, and nucleotides, to which it would otherwise not have access [60]. Consequently, in states of deprivation, autophagy prevents tumor cells from undergoing apoptosis. This may suggest that in a tumor, autophagy maintains tumor cells when restricted angiogenesis leads to hypoxia and nutritional deprivation, and depending on circumstances, can be treated through a common form of autophagy. The mechanism by which autophagy prevents tumor growth is not certain. The removal of damaged mitochondria and other organelles may limit the growth of tumor cells or reduce mutagenesis or other damage caused by reactive oxygen species. Alternatively, autophagy may destroy tumor cells that are still proliferating [61].

## Molecular crosstalk between LncRNAs and autophagy pathways in osteosarcoma

**Fig. 2:**
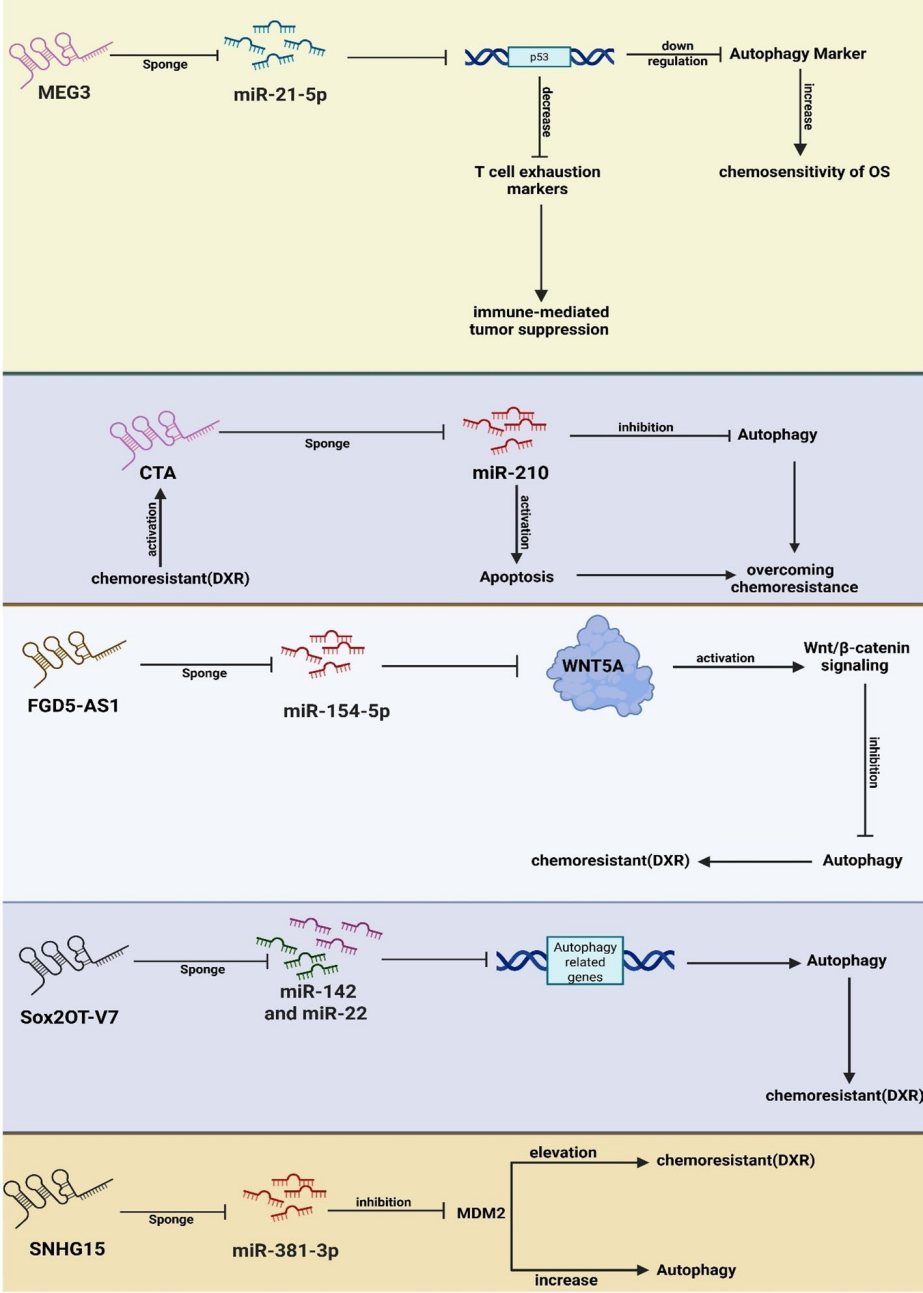
LncRNAs regulate autophagy pathways implicated in chemoresistance in osteosarcoma

**Fig. 3:**
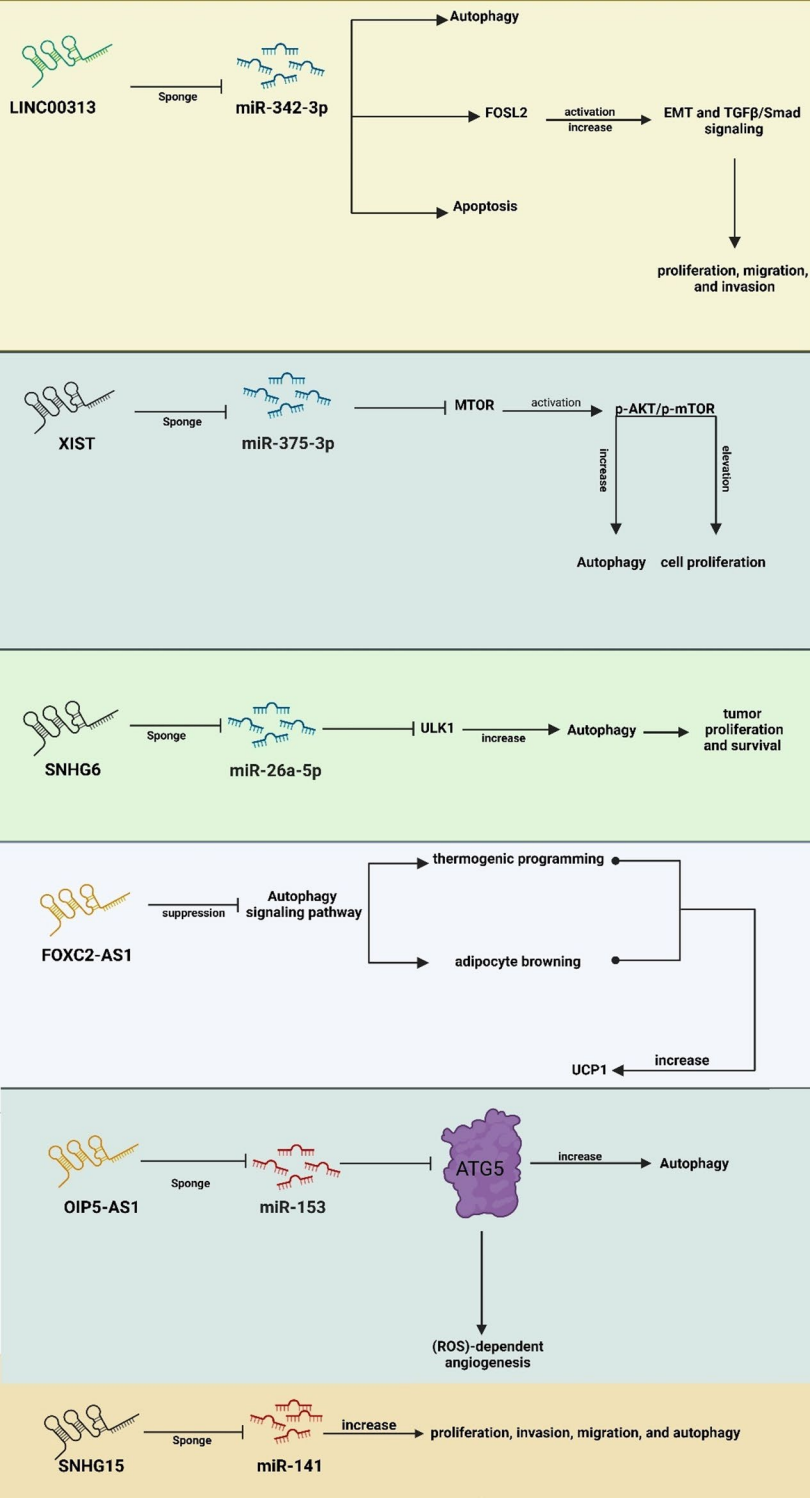
The regulatory activities of diverse lncRNAs in OS. The main function of lncRNAs is mediated by their interaction with certain miRNAs and downstream signaling pathways that regulate autophagy, cell proliferation, apoptosis, invasion, and migration

Several mechanisms regulate autophagy, and one of the more recently recognized mechanisms is the role of ncRNAs [62]. LncRNAs may regulate the expression of protein-coding genes in both positive and negative directions through their interactions with RNA, protein, and chromatin structures [63]. The expression levels of autophagy-related lncRNAs predict the prognosis in patients with various types of cancers [64–66]. In the subsequent sections, we will describe several lncRNAs, emphasizing their mechanistic roles in the regulation of autophagy during OS progression. Figure 2 demonstrates the role of several lncRNAs in chemoresistance through autophagy regulation in OS. We highlight the intricate networks through which these lncRNAs (Table 1) interact with miRNAs and essential signaling pathways, ultimately influencing tumor cell survival, proliferation, and responsiveness to therapy (Fig. 3).

### LncRNA MEG3

LncRNA maternally expressed gene 3 (lncRNA MEG3) is approximately 1.6 kb in size. Many cancers, including OS, have been found to exhibit aberrant expression of the lncRNA MEG3 [67]. Recent findings indicate that lncRNA MEG3 may significantly influence tumor immunity and chemoresistance, two key factors affecting OS growth and therapeutic response.

The tumor immunological microenvironment (TIME) is crucial in tumorigenesis and cancer progression, influencing overall treatment and survival outcomes. Due to TIME importance, immunotherapy strategies have surfaced as a viable strategy for bone and soft tissue sarcomas [68, 69]. Nonetheless, immune-related lncRNA indicators in OS remain little investigated. Initial studies indicate that lncRNA MEG3 expression correlates with immune cell infiltration and prognosis in OS [70]. According to a study by Huang et al. lncRNA MEG3 was significantly overexpressed in OS cell lines, while the expression of miR-21-5p was downregulated correspondingly [70]. Previous studies have confirmed that lncRNA can act as miRNA sponges and subsequently regulate the function of miRNA. To predict the potential miRNA target of lncRNA MEG3, Huang et al. conducted a bioinformatic analysis using the TargetScan database, which revealed that lncRNA MEG3 shares the response element with miR-21-5p. The study demonstrated through Western blot analysis that the pCDNA-MEG3 + miR-21-5p mimic group showed significantly decreased p53 expression compared to the pCDNA-MEG3 group, while the pCDNA-MEG3 + miR-21-5p inhibitor group exhibited significantly increased p53 expression. This bidirectional modulation confirms the regulatory axis where MEG3 functions as a molecular sponge for miR-21-5p, thereby liberating p53 from miRNA-mediated suppression [70]. Notably, MEG3 expression itself is under epigenetic control through DNA methylation, as previous studies have demonstrated that hypermethylation of the MEG3 promoter region leads to loss of MEG3 expression in various cancers. The expression of MEG3 can be restored through demethylation using DNMT inhibitors, suggesting a reversible epigenetic mechanism controlling this lncRNA. Furthermore, a potential feedback loop exists in this regulatory network, as p53, once activated, can stimulate p53-mediated transcriptional activation and selectively activate p53 target genes, which may include genes that influence the epigenetic machinery controlling MEG3 expression, thereby creating a self-reinforcing tumor suppressor circuit [70].

Moreover, upregulation of lncRNA MEG3 lowered levels of autophagy markers, such as Beclin 1 and LC3B, and reduced cancer cell response to treatment. Autophagy contributes to both promoting and inhibiting chemoresistance, and seems to be negatively regulated by lncRNA MEG3 in OS cell lines. Autophagy has a dual role in cancer progression; mutant p53, together with oncoproteins, inhibits autophagy and facilitates excessive protein accumulation in starved/stressed cancer cells, while excessive autophagy may also induce autophagic programmed cell death or apoptosis. The complexity of the feedback loop emerges as mutant p53 can inhibit autophagy as well as subsequently complete restoration of wild-type p53 through induction of MEG3 to improve apoptosis and demonstrate a net decrease in autophagy, which may depend on the situation of the cellular context. The results of the study demonstrated significant positive correlations between LC3B and markers associated with T cell exhaustion, TIM-3, CTLA4, and GZMB, suggesting that autophagy may play a role in immune evasion mechanisms associated with T cell exhaustion in bone and soft tissue sarcoma. Further investigation showed that lncRNA MEG3 acted as a sponge for miR-21-5p to modulate TP53 expression, as overexpression of lncRNA MEG3 resulted in elevated levels of TP53 [70]. Analysis of the GEPIA database validated the existence of a significantly positive association between lncRNA MEG3 and TP53. The MEG3-p53 Pathway involves epigenetic regulation at multiple levels: (1) the methylation status of the MEG3 promoter determines MEG3 expression, and (2) MEG3 expression regulates p53 through the miR-21-5p axis. Once activated p53 can suppress the expression of the gene that encodes MDM2 (and its protein product), an E3 ubiquitin ligase that typically promotes p53 degradation. This sets up a regulatory feedback loop where MEG3 suppresses MDM2 expression, allowing p53 to accumulate, translocate into the nucleus, and activate p53-dependent transcription. TP53 also exhibited significant negative correlations with the infiltrating levels of macrophages and dendritic cells, and negative correlations with markers of T cell exhaustion TIM-3 and GZMB. This multi-level regulation demonstrates crosstalk between the MEG3/miR-21-5p/p53 axis and TME as p53 restoration impacts immune cell infiltration and states of functional exhaustion through epigenetic remodeling of TME. Accordingly, previous studies demonstrated that TP53 was strongly linked to lower levels of T cell exhaustion markers in bone and soft tissue sarcomas. Both Beclin 1 and LC3B demonstrated notable positive correlations with the level of CD8 + T cell infiltration, whereas lncRNA MEG3 displayed negative correlations with these autophagic markers. This indicates a regulatory interaction that MEG3 overexpression both inhibits autophagy and influences the actions of immune cells, although LC3B could be actively promoting TIM-3-mediated T cell exhaustion. The epigenetic silencing of MEG3 via promoter hypermethylation due to DNA methyltransferase 1 (DNMT1) plays an important role in how cancer cells evade tumor suppression, as demethylating RNA MEG3 restores MEG3 expression, thereby reversing the inhibition of the p53 pathway and promoting anti-tumor immunity. The results from the xenograft tumor model further corroborate this mechanism, demonstrating that the DDP + pCDNA-MEG3 combination group (treated with cisplatin + MEG3 overexpression) significantly reduced tumor weight and reduced the abundance of markers of T cell exhaustion (TIM-3, LAG3, GZMB) compared with the DDP (cisplatin only) group. This evidence indicates that MEG3 mitigates T cell dysfunction and augments immune-mediated tumor suppression via the MEG3/miR-21-5p/p53pathway [70]. These results were further supported by studies from immunogenomic databases showing that lncRNA MEG3 can affect T cell fatigue by regulating autophagy, leading to tumor suppression [71]. As a result, enhancing the expression of lncRNA MEG3 might make OS more responsive to chemotherapy by promoting anti-cancer immune response through the miR-21-5p/p53 pathway and autophagy. The data indicate that lncRNA MEG3 may serve as a potential therapy for overcoming OS chemoresistance [70].

### LncRNA FGD5-AS1

LncRNA FGD5-AS1 is overexpressed in OS, and its upregulation predicted poor prognosis for the patient [72]. Fei et al. demonstrated that lncRNA FGD5–AS1 is highly expressed in DXR-resistant OS cells, and lncRNA FGD5‐AS1 knockdown prevents cancer cell growth, invasion, and autophagy. LncRNA FGD5‐AS1 directly interacts with miR‐154‐5p and sponges it, leading to upregulation of its downstream target WNT5A [73].

This regulatory axis exhibits functional crosstalk, as WNT5A signaling can modulate the expression of FGD5-AS1 by transcriptional feedback, creating a synergistic loop that reinforces drug resistance. Furthermore, epigenetic modifications such as DNA methylation and histone modifications (e.g., acetylation) at the promoter region of FGD5-AS1 may also regulate FGD5-AS1 depending on exposure to chemotherapeutic stresses, adding another dimension to this pathway [73]. The Wnt/β-catenin pathway is central in cancer growth, invasion, and drug resistance. The Wnt/β-catenin signaling is very important for the growth, invasion, and treatment resistance of cancer. The WNT5A is highly expressed in OS and can promote cancer progression by increasing tumor cell proliferation and invasion [74, 75].

Aside from its traditional function, WNT5A is capable of activating non-canonical Wnt signaling pathways, such as the Wnt/Ca²⁺ pathway and the Wnt/PCP pathway, and may have independent functions in regulating autophagy and also chemoresistance, through a variety of downstream effectors, such as CaMKII and JNK. Numerous studies illustrate a connection between the Wnt pathway and autophagy, with activation of Wnt signaling and an increase in β-catenin levels leading to an inhibition of autophagy. However, with respect to doxorubicin (DXR) resistance, WNT5A appears to promote protective autophagy in resistant cells instead of inhibiting it. This finding indicates a potential context-dependent switching between canonical Wnt signaling outputs and non-canonical Wnt signaling outputs. This switching capacity can also be dependent on the cellular metabolic state, as well as the Wnt receptors and co-receptors that exist in the resistant cells. Many studies demonstrate a link between the Wnt pathway and autophagy, as activation of Wnt signaling and increased levels of β-catenin can suppress autophagy [76]. MiR-154‐5p participates in the pathogenesis of various diseases and is investigated in multiple cancer types. Previous studies reported that miR‐154‐5p was suppressed in multiple cancer types, where it acted as a tumor suppressor [77, 78]. However, the function of miR‐154‐5p in chemotherapy resistance was rarely investigated previously. In breast cancer, miR‐154 suppresses nicotinamide phosphoribosyl transferase and enhances tumor cells’ susceptibility to DXR [78]. DXR and methotrexate are two major drugs used to treat OS [79]. Fei et al. also found that miR‐154‐5p is associated with DXR resistance in OS. Knocking down miR-154-5p made OS cell lines less sensitive to DXR and increased their proliferation, migration, and autophagy [73].

However, these findings were limited to two cell lines (HOS and MG-63), and the artificially generated DXR-resistant models may not fully recapitulate clinical drug resistance mechanisms. In this study, WNT5A was identified as a downstream target of the lncRNA FGD5-AS1/miR‐154‐5p axis and acted as an oncogene promoting drug resistance in OS. Altering WNT5A expression diminished the effects of FGD5‐AS1 knockdown or miR‐154‐5p overexpression in DXR-resistant OS cells. Although the nude mouse xenograft model is an important demonstration of in vivo relevance, it is limited by small sample sizes, subcutaneous, rather than orthotopic, implantation, dependence on overexpression-based rescue, rather than CRISPR knockout or pharmacological inhibition, and, critically, absence of clinical samples to correlate expression levels with patient treatment responses. This study demonstrated a significant role of lncRNA FGD5‐AS1 in OS progression and DXR-resistance through regulating the Wnt/β-catenin pathway via miR-154-5p [73]. Future work for validation can include patient-derived models, larger statistically powered cohorts, and multi-omics approaches to validate basin regulatory mechanisms.

### LncRNA CTA

miR-210 is a well-known oncogenic miRNA with aberrant expression in different types of cancer cell lines [80]. In gamma-irradiated conditions, miR-210-overexpressing non-small cell lung cancer cells can proliferate and have reduced caspase-3 activity, resulting in decreased apoptosis [81]. In OS, miR-210 overexpression strongly correlates with tumor development; thus, Wang et al. screened OS cell lines to identify all lncRNAs that regulate the expression of miR-210. The result showed that expression of lncRNA CTA significantly correlated with miR-210 function specifically in XR-resistant cells. LnRNA CTA acts to reduce the expression levels of miR-210, as demonstrated in our previous studies. Mechanistically, lncRNA CTA acts as a competing endogenous RNA (ceRNA) because it has miR-210 binding sites in its 3’-UTR and allows for competitive sequestration of miR-210 away from its mRNA targets, establishing a post-transcriptional control layer that buffers miR-210 activity. This ceRNA mechanism establishes a molecular titration system whereby lncRNA CTA expression levels dictate the pool of available miR-210 for autonomously targeting genes. In fact, we show that DXR activated lncRNA CTA and that lncRNA CTA sequestered miR-210 from its targets, leading to reduced miR-210 expression, which inhibited autophagy and induced apoptosis. This regulatory axis coordinates complex pathway crosstalk: miR-210 targets are released, triggering co-derepression of multiple pro-apoptotic factors (Casp8ap2, AIFM3), while at the same time, the autophagy machinery is deregulated due to altered lipidation of LC3-I to LC3-II, reduced autophagosome formation, and decreased expression of autophagy receptors BNIP3/BNIP3L. The simultaneous regulation of Bcl-2 family proteins hits a key juncture in which inhibited autophagy combines with enhanced mitochondrial outer membrane permeabilization (MOMP) to exacerbate apoptosis. Additionally, the reciprocal regulation of lncRNA CTA and miR-210 suggest that a feedback loop is at play: the increased expression of lncRNA CTA in the face of DXR treatment may involve stress-induced transcription factors which bind to indicated regions of the CTA promoter region, while lncRNA CTA acts to inhibit expression of miR-210, the sequestration of miR-210 prevents its negative feedback on upstream signaling pathways, including potential HIF-1α stabilization under hypoxic tumor microenvironments. Wang et al. found that lncRNA CTA promotes autophagy deactivation in response to DXR treatment, leading to enhanced sensitivity and response to DXR. Furthermore, lncRNA CTA increased the expression of Caspase-8-associated Protein 2 (Casp8ap2) and Apoptosis-Inducing Factor, Mitochondrion-Associated 3 (AIFM3), which are downstream targets of miR-210, indicating its effect on autophagy and apoptosis via miR-210sponge. This molecular architecture of a regulatory network is not straightforward, as Casp8ap2, once released from repression, can facilitate death receptor-mediated extrinsic apoptotic signaling by scaffolding caspase-8 activation, while AIFM3 contributes to mitochondrial dysfunction and activates caspase-independent cell death pathways. This duality of mechanism, which can activate the apoptotic process through caspase-dependent and caspase-independent pathways, while impairing cytoprotective autophagy all at the same time, allows for a multi-faceted assault on cancer cell survival. Supporting this idea of the convergence of these pathways is the concurrent alteration of P62/SQSTM1, which, while serving as an autophagy adaptor protein, also pharmacologically regulates apoptosis by interacting with caspase-8, suggesting a biochemical bridge connecting autophagy flux and apoptotic commitment. Further, it is important to highlight the epigenetic aspect, exposure to DXR may trigger chromatin remodeling at the CTA locus involving global histone modification or changes in DNA methylation, perhaps through stress-activated kinases (i.e., p38 MAPK, JNK) which would phosphorylate transcription factors binding the CTA promoter, enabling feed-forward dynamics with treatment response. These findings show that lncRNA CTA is essential for DXR-induced apoptosis, and targeting the lncRNA CTA/miR-210 signaling pathway can help overcome OS chemoresistance [82].

### LncRNA Sox2OT-V7

The SOX2 overlapping transcript (SOX2OT), a lncRNA, includes the pluripotency gene SOX2 and generates eight transcript variants (V1–V8), exhibiting variant-specific expression in various organs and malignancies [83]. Among them, SOX2OT variant 7 (V7) has emerged as a potential oncogenic player in OS, though its specific role remained unclear. It has emerged as a significant factor in DXR-induced autophagy in OS. LncRNA SOX2OT-V7 downregulation correlates with decreased autophagy markers and LC3 localization, confirming its role in autophagy regulation. Additionally, lncRNA SOX2OT-V7 impacts cancer stemness through the SOX2 pluripotency axis, which is crucial for OS chemoresistance [83–86]. Treatment with epigallocatechin gallate (EGCG) (a green tea polyphenol with antitumor properties) significantly downregulated lncRNA SOX2OT V7 in a dose-dependent manner. When combined with DXR, EGCG synergistically enhanced cell growth inhibition, as confirmed by the coefficient of drug interaction (CDI) analysis. EGCG treatment not only reduces self-renewal of osteosarcoma stem cells (OSCs) but also amplifies DXR’s inhibitory effect on OSC proliferation via inhibiting the Notch pathway. The lncRNA Sox2OT-V7 overexpression enhances Notch signaling by upregulating downstream targets Hes1 and Hey1. Further qRT-PCR analyses revealed that among Notch receptors and ligands, only Notch3 and DLL3 were directly affected by lncRNA Sox2OT-V7 in OSCs, therefor inhibitory effect of EGCG on OSCs via lncRNA SOX2OT-V7 is partly mediated by suppression of the Notch3/DLL3 signaling axis [85]. Zhu et al. in a recent study, showed that lncRNA Sox2OT-V7 is significantly overexpressed in OS tissues and cell lines, especially in DXR-resistant samples, further highlighting its critical involvement in chemoresistance. While this does lay a foundation for a distinct relationship, the upstream drivers of Sox2OT-V7 overexpression in chemoresistant cells remain open questions. Sox2OT-V7 expression in resistant cells may be maintained via a positive feedback loop, which could be induced by DXR-mediated cellular stress responses (e.g., NF-κB or p53 pathways) where they bind the Sox2OT promoter and transactivate transcription, potentially maintaining the cell in a sustained high-autophagy chemoresistant state. OS cells, in response to DXR treatment, can increase pro-survival autophagy capacity as well as lncRNA Sox2OT-V7 expression, which initiates DXR-resistance development. The activation of autophagy itself may also epigenetically regulate Sox2OT-V7 expression. Autophagy may affect the pool of metabolites such as α-ketoglutarate, which can modulate the activity of histone demethylases (e.g., KDM5A/B), modulating the chromatin context at the Sox2OT locus to transcriptionally enhance Sox2OT-V7 transcription. LncRNA Sox2OT-V7 functions as a ceRNA, sequestering tumor-suppressive miRNAs (miR-142 and miR-22) that exhibit a negative correlation with its expression in OS. It is inherently important to acknowledge that these mechanistic insights are based, for the most part, on the U2OS cell line in addition to its xenografts. It is still unclear if this axis generalizes to the known heterogeneity of human OS, given that the model does not account for intra-tumoral heterogeneity or the native tumor microenvironment. Additionally, even though the clinical correlation is significant, it is drawn from a limited clinical cohort [87]. Both miRNAs are recognized autophagy inhibitors and directly target key autophagy regulators: miR-142 targets ULK1, ATG4A, and ATG5, whereas miR-22 targets ULK1, consequently impeding the onset and development of autophagy. MiR-22 increases 5-fluorouracil sensitivity in colorectal cancer by suppressing autophagy, whilst miR-142 targets autophagy-related genes (ATGs) such as ATG5 and ATG16L1, thereby sensitizing hepatocellular carcinoma to sorafenib [88, 89]. The lncRNA Sox2OT-V7 knockdown markedly diminished DXR-induced autophagy, a result that is partially mitigated by the downregulation of miR-142 or miR-22, through the lncRNA Sox2OT-V7/miR-142/miR-22 regulatory axis. Inhibition of lncRNA Sox2OT-V7 reduces ATG gene expression and disrupts autophagic flow, as evidenced by alterations in the LC3-II/I ratio, Beclin-1, and p62 levels, with these effects counteracted by miRNA inhibition. Collectively, lncRNA Sox2OT-V7 acts as a pro-oncogenic and chemoresistance-enhancing factor by participating in the initial development of resistance and augmenting DXR-induced autophagy. The Sox2OT-V7/miR-142/miR-22/ATG pathway signifies a new mechanism of OS chemoresistance and a potential therapeutic target [87].

### LncRNA SNHG15

Small nucleolar RNAs (snoRNAs) are a class of ncRNAs involved in regulating other RNA functions. They are involved in the chemical modification of RNAs and the processing and stability of RNAs [90]. SnoRNAs typically contain 60–300 nucleotides, therefor can be categorized as short or long ncRNAs. Small nucleolar RNA host gene 15 (SNHG15) is an lncRNA with oncogenic effects in several types of cancers [91, 92]. Two prominent studies investigated the roles and effects of lncRNA SNHG15 in OS. Among all putative targets of lncRNA SNHG15 in OS cell lines, miR-141 and miR-381-3p are closely regulated by lncRNA SNHG15 in osteosarcoma cell lines [93, 94]. MiR-141 is a member of the miR-200 family with various effects in cancer progression [95]. According to Liu et al., miR-141 exerts tumor-suppressive effects in OS cell lines, and lncRNA SNHG15 directly suppresses its expression, diminishing its tumor-suppressive effects. Overexpression of miR-141 decreased OS cell proliferation, invasion, and autophagy, while suppression did the opposite. These data suggest that lncRNA SNHG15 negatively regulates miR-141 to promote OS growth [93]. Zang et al. showed that miR-381-3p is another target of lncRNA SNHG15 associated with its role in enhancing OS progression through regulating autophagy [94].

This was demonstrated in vitro with two DXR-resistant humanOS cell lines (U2OS/DXR and MG63/DXR) and an animal xenograft model (*n* = 5 per group), although the in vitro data need to be confirmed in a larger number of cell lines, as well as primary patient-derived cultures; this will enhance the generalizability of our study. For human tissue samples, the sample size (*n* = 30 per group) was large enough for the first correlation analyses, although confirmation of clinical relevance would require larger multi-center cohorts. LncRNA SNHG15 promotes proliferation, autophagy, and XXR resistance in OS cells via regulating the miR-381-3p/GFRA1 axis [94]. GFRA1, also known as GDNF family receptor alpha 1, is a co-receptor involved in the activation of key cellular signaling pathways such as PI3k and MAPK. GFRA1 plays an important role in enhancing chemoresistance in OS [96]. It increases DXR resistance in OS cell lines by inducing autophagy. Based on Zhang et al., lncRNA SNHG15 upregulates GFRA1 expression in OS cell lines via sponging miR-381-3p, leading to the development and progression of DXR resistance in OS cell lines. However, functional rescue assays based on GFRA1 elevation in SNHG15-downregulated cells might validate the axis. In addition, the small number of autophagy inhibitors tested (3-MA) is a limitation to understanding the mechanisms in depth, and other strategies, such as LC3 flux assays or electron microscopy analysis, could reinforce those autophagic conclusions [94].

### LncRNA OIP5-AS1

Alongside autophagy, organogenesis is a crucial process in the advancement of OS. It accelerates conditions for fast-growing tumor cells to receive oxygen and nutrients, which promotes tumor growth and spread [17, 97]. In addition, autophagy can enable immune evasion of cancer cells and enhance cancer cell metabolism during tumor growth [98, 99]. ATG5 is pivotal in autophagosome formation and regulates mitochondrial activity, cell proliferation, apoptosis, and adhesion. The downregulation of ATG5 impedes autophagy and decreases the proliferation of skin fibroblast cells, indicating its potential as a therapeutic target [100]. The lncRNA OIP5-AS1, which is markedly overexpressed in OS tissues and cells, has been shown to have an oncogenic effect in recent research [101, 102]. Increased expression of lncRNA OIP5-AS1 is associated with unfavorable prognosis in OS patients, with a notable reduction in 5-year survival rates [103]. This indicates its possible value as a prognostic biomarker. LncRNA OIP5-AS1 promotes OS cell proliferation, invasion, and resistance to apoptosis, partly through its ability to function as a ceRNA [102]. It exerts its regulatory effects by sponging multiple miRNAs, including miR-340-5p, miR-137-3p, miR-377-3p, and notably, miR-153 [101, 102, 104, 105]. MiR-153, situated on chromosome 6, is associated with oxidative stress response, cisplatin resistance, and proliferation in OS cells [106, 107]. The lncRNA OIP5-AS1/miR-153/ATG5 axis involves lncRNA OIP5-AS1 sponging miR-153, alleviating its inhibitory effects on ATG5, thereby increasing ATG5 expression and facilitating autophagy. DNA methylation and histone modifications must support the fine-tuning of lncRNA OIP5-AS1, miR-153, and ATG5 transcription, resulting in autophagy modulation with OS. As for OIP5-AS1, its promoter regions may attract epigenetic enzymes (e.g., histone methyltransferases (like EZH2) and acetyltransferases (like P300)) to mediate specific modifications or alterations of the histone marks (such as H3K27me3, repressive; H3K4me3, activating), resulting in silencing or augmenting its expression. The same processes could be acting to mediate between miR-153 and ATG5, either through DNA methylation of CpG islands on promoters or histone modification changes that directly affect the transcriptional status of these miRNAs and autophagy-related target molecules [105].

This occurs through the activation of the ULK1 complex and the PI3K pathway, resulting in the creation of the ATG12–ATG5–LC3 complex and the onset of autophagy [108]. Furthermore, ULK1 is also known to be activated downstream of AMPK, which integrates energy stress signals, and mTOR acts in autophagy inhibition. 

OIP5-AS1/miR-153/ATG5 activity may dynamically respond to metabolic stimuli. Feedback loops can be observed with increased autophagy being able to regulate OIP5-AS1 and miR-153 levels, hence either stabilizing or inhibiting pathway activity leading to cellular homeostasis. Furthermore, Li et al. showed that exosomal lncRNA OIP5-AS1 can also affect angiogenesis in OS. Exosomal secretion of LncRNA OIP5-AS1 improved its transfer to endothelial cells and promoted apoptosis through regulating the miR-153/ATG5 axis. These findings revealed that ATG5 modulates angiogenic activities in endothelial cells through regulating reactive oxygen species (ROS)-dependent mechanisms [105]. The downregulation of exosomal lncRNA OIP5-AS1 decreased angiogenesis, underscoring its potential as a therapeutic target for inhibiting tumor vascularization. Moreover, ATG5-mediated autophagy in OS has been associated with critical oncogenic pathways, including NF-κB and p53/Rb, but the function of lncRNA OIP5-AS1 in regulating these pathways is yet to be thoroughly clarified. Considering that autophagy facilitates immune evasion, the lncRNA OIP5-AS1/miR-153/ATG5 axis may influence OS tumor immunology. This necessitates additional examination of its influence on immune surveillance and response inside the tumor microenvironment. In conclusion, exosomal lncRNA OIP5-AS1 is crucial in OS by concurrently enhancing autophagy and angiogenesis via the regulation of the miR-153/ATG5 axis. These functions facilitate tumor proliferation, endurance, and possible resistance to therapy. OIP5-AS1 presents considerable potential as a diagnostic, prognostic, and therapeutic target in OS [105].

### LncRNA XIST

Despite significant advancements in the identification of molecular regulators in OS, the complex lncRNA–miRNA–mRNA regulation networks implicated in OS progression are nonetheless only partially comprehended. LncRNA XIST is a well-studied lncRNA with oncogenic effects in different types of cancers [109, 110]. Investigations revealed significant upregulation of lncRNA XIST and downregulation of miR-375-3p in OS tissues and cell lines. Bioinformatics analysis indicated, and luciferase reporter experiments validated, that miR-375-3p is a direct target of lncRNA XIST. Moreover, mTOR was recognized as a downstream target of miR-375-3p, indicating a regulatory axis of lncRNA XIST/miR-375-3p/mTOR that plays a role in OS pathogenesis. LncRNA XIST knockdown in OS cells led to decreased expression of LC3-I and LC3-ΆΆ protein levels, and increased p63 protein levels, further showing that lncRNA XIST/miR-375-3p/mTOR axis participates in OS progression through regulating autophagy [111]. AKT’s downstream rapamycin (mTOR) is crucial for the AKT/mTOR signaling pathway. The AKT/mTOR signaling plays a key role in tumorigenesis via regulating cell autophagy, cell cycle progression, and apoptosis in various cancers such as prostate cancer, endometrial cancer, and OS. Sun et al demonstrated the key effect of AKT/mTOR in OS progression, as they found that caffeine exerts tumor-suppressive effects in OS via inhibiting AKT/mTOR/S6K, NF-kB, and MAPK pathways, leading to enhanced apoptosis and tumor suppression [111].

Caffeine and/or its downstream inhibition pathways could affect gene expression of key genes through changes in histone modifications and/or DNA methylation, and other factors sustaining the apoptotic phenotype. Chromatin immunoprecipitation (ChIP) assays for the presence of histone marks at the promoters of NF-κB target genes or RNA-seq for the identification of dysregulated ncRNAs (lncRNAs, circRNAs, etc.) would also be an important future study for understanding this NF-κB-related network of factors. The findings can be further validated for their generalizability in the OS cell lines (U2OS, Saos-2, MG-63) panel and patient-derived primary cells. Likewise, the NF-κB pathway inhibition could also be confirmed by electrophoretic mobility shift assays (EMSA) or more direct transcriptional activity assays, such as a reporter gene assay. Rescue assays, such as utilizing a constitutively active form of AKT or MEK, would potentially also show necessity and sufficiency for each of these pathways in mediating caffeine’s effects [112–114]. In summary, Sun et al. showed that lncRNA XIST promotes OS progression by sequestering miR-375-3p, resulting in the upregulation of mTOR and the activation of the AKT/mTOR signaling pathway, which jointly enhances autophagy and cell proliferation while suppressing apoptosis. Targeting the XIST/miR-375-3p/mTOR pathway may provide a unique therapeutic strategy to address autophagy-mediated chemoresistance and unchecked proliferation in OS [111].

### LncRNA SNHG6

Small Nucleolar RNA Host Gene 6 (SNHG6) is an oncogenic lncRNA that exerts its effects mostly through sponging tumor-suppressive miRNAs like miR-141 and miR-101-3p [93, 115]. In OS tissues and cell lines, lncRNA SNHG6 is highly expressed compared to normal tissues. High lncRNA SNHG6 expression is associated with advanced TNM stage, deeper invasion, and worse prognosis, suggesting a key involvement in OS pathogenesis. Functional investigations showed that lncRNA SNHG6 activates OS cell proliferation, migration, and invasion, while its silencing reduces cell viability, causes G0/G1 cell cycle arrest, and enhances apoptosis. Importantly, inhibiting SNHG6 also drastically reduces OS cell autophagy, suggesting a dual function in tumor development and survival. Zhu et al. showed that lncRNA SNHG6 acts as a miR-26a-5p sponge, which is a tumor-suppressive miRNA and regulates autophagy in various malignancies [116]. ULK1 is a serine/threonine kinase that plays a key role in autophagosome formation and autophagy initiation [117]. Several studies demonstrated that ULK1 is one of the downstream targets of miR-26a-5p. MiR-26a-5p regulates ULK1 in cardiac fibroblasts, and its overexpression increases apoptosis and chemotherapy sensitization in hepatocellular carcinoma cells by suppressing ULK1 [118]. Zhu et al. showed that miR-26a-5p directly targets ULK1 in OS cells, suppressing autophagy. LncRNA SNHG6 promotes upregulates of ULK1 expression via miR-26a-5p sponge and reduces its inhibitory effect on ULK1. This shows that the lncRNA SNHG6/miR-26a-5p/ULK1 regulatory axis is involved in OS progression via regulating autophagy. This study is the first to show that SNHG6 is involved in the miR-26a-5p/ULK1 pathway in OS. Since ULK1 initiates autophagy, SNHG6 silencing-induced miR-26a-5p reduction is a possible therapeutic method. Zhu et al. also found that lncRNA SNHG6 knockdown increased the expression of pro-apoptotic proteins such as Caspase-3 and ATF3, which regulate programmed cell death. This suggests that lncRNA SNHG6 knockdown affects both autophagy and caspase-dependent apoptosis, which may improve chemotherapeutic efficacy in resistant OS cells [116].

### LINC01410

Long intergenic non-coding RNA (LINC) is a type of lncRNA that is transcribed from intergenic regions and participates in different cellular processes, such as gene expression regulation and miRNA sponge [119]. Recent investigations have shown a comparable tumor-promoting function of LINC01410 in OS. LINC01410 was shown to be overexpressed in OS tissues and cell lines, and its aberrant expression promoted cell invasion and proliferation by modulating miR-3128 in OS. Furthermore, the overexpression of LINC01410 enhanced the invasion and proliferation of OS cells by regulating miR-3128. MiR-3128 was found to be downregulated in OS cells; however, silencing LINC01410 expression resulted in upregulation of miR-3128, indicating its function as a miRNA sponge. Xu et al. showed that LINC01410 promotes OS progression and may represent a novel therapeutic option for OS [120]. The research showed that LINC01410 was overexpressed in OS specimens and cell lines. A significant increase in expression was identified in 73% of OS patients when compared to normal controls. LINC01410 acted as a ceRNA by sponging miR-3128 to increase OS cell proliferation, cell cycle progression, and invasion. In addition to the classical ceRNA activity, the study identified more complexity in the regulatory network of LINC01410. An inverse relationship between LINC01410 and miR-3128 expression was observed in OS patients, suggesting a reciprocal feedback relationship. In addition, LINC01410 overexpression modified epithelial-mesenchymal transition (EMT) markers, which induced N-cadherin and Vimentin expression and suppressed E-cadherin, suggesting crosstalk with EMT signaling pathways other than mere miRNA sponging. Additionally, functional rescue experiments demonstrated that miR-3128 overexpression could reverse the positive effects of LINC01410 on cell cycle progression and on proliferation markers (Ki-67 and cyclin D1), EMT markers, and invasive potential, thus confirming the bidirectional regulatory axis. The multi-layered mechanistic framework of LINC01410, which includes, but is not limited to, ceRNA activity, modulation of EMT pathways, and feedback, positions LINC01410 as a central node in OS pathogenesis, rather than as a linear regulator, emphasizing its potential as a targeted therapy. The research conducted singular time-point analyses and did not have longitudinal follow-up data relating LINC01410 expression to clinical outcomes, including overall survival, progression-free survival, and/or metastatic potential. In terms of clinical translation, LINC01410 may be a promising diagnostic biomarker and therapeutic target; however, significant practical obstacles need to be mitigated before clinical translation. A considerable limitation in developing an effective drug delivery system that selectively targets LINC01410 in OS cells exists; systemic delivery of antisense oligonucleotides or small interfering RNAs faces challenges induced by factors including rapid degradation, poor cellular penetration, and limited tumor penetration. Nanoparticle delivery platforms, lipid nanoparticles, and/or exosome-mediated delivery systems may provide solutions; however, these will need extensive optimization and assessment [120].

### LINC00313

LINC00313 has recently been related to several malignancies, including lung cancer and papillary thyroid carcinoma, where it is persistently associated with an unfavorable prognosis [121, 122]. In a recent study, Chen et al. examined the functional role and molecular mechanism of LINC00313 in OS, including its interaction with miR-342-3p and the downstream target FOSL2, which contribute to the progression and metastasis of OS. LINC00313 was identified as highly elevated in OS tissues and cell lines relative to normal counterparts. Elevated expression exhibited a favorable correlation with tumor size, TNM stage, distant metastasis, and reduced overall survival, indicating its potential as a prognostic biomarker. Kaplan-Meier survival analysis revealed a pronounced disparity in 5-year survival rates, indicating that patients with low levels of LINC00313 exhibited significantly improved outcomes compared to those with high expression, aligning with findings noted in lung cancer and papillary thyroid carcinoma [123]. Moreover, miR-342-3p has been demonstrated to function as a tumor suppressor and is downregulated in several malignancies [124]. Prior studies demonstrated that miR-342-3p inhibits the proliferation, migration, and invasion of OS cells [125]. According to Chen et al. LINC00313 functions as a ceRNA, sponging miR-342-3p. In OS tissues, miR-342-3p was markedly downregulated, while its overexpression suppressed oncogenic traits and induced autophagy and apoptosis in OS cell lines (U2OS and MG-63). Further investigation revealed that the tumor-suppressive effects of miR-342-3p in OS were associated with downregulating FOSL2 expression [123]. FOSL2 (Fos-like antigen 2 or Fra-2) is a transcription factor that participates in various biological processes, including cancer initiation and progression. downregulation of FOSL2 has been demonstrated to exert anti-tumor effects via inhibiting epithelial-mesenchymal transition (EMT), migration, and invasion through regulating TGFβ/Smad signaling [126]. In summary, LINC00313 promotes OS progression by acting as a molecular sponge for miR-342-3p, thereby decreasing FOSL2 expression. LINCC00313 knockdown impedes OS cell proliferation, migration, and invasion, while enhancing apoptosis and autophagy. The LINC00313/miR-342-3p/FOSL2 axis thus forms a functional circuit through which LINC00313 contributes to OS malignancy. The recognition of FOSL2 as a functional effector in this axis underscores the carcinogenic capacity of LINC00313 through the regulation of essential transcriptional regulators and signaling pathways [123]. Additionally, the LINC00313/miR-342-3p/FOSL2 axis provides a molecular framework for studying gene regulation networks, tumor microenvironment interactions, and signaling pathways like TGFβ/Smad and NF-κB.

### LncRNA NEAT1

NEAT1 resides in the human chromosome 11q13.1 locus and associates with RNA-binding proteins to generate the essential structural scaffolds of paraspeckles [127, 128]. NEAT1 contains two transcript variants sharing a common transcription initiation site, the shorter transcript variant NEAT1-1 (MENε, 3.7 kb) and the longer transcript variant NEAT1-2 (MENβ, 22.7 kb) [129]. NEAT1-2 is the common structural unit of the paraspeckle, in which the central domain consists of several binding sites that are engaged in paraspeckle-associated protein interactions, such as SFPQ, NONO, PSPC1, and FUS. The presence of these binding sites is required and sufficient for paraspeckle formation, stability, and structural integrity.

HMGB1, a high mobility group protein superfamily member, was identified as a promoter of radiotherapy and chemotherapy resistance in cancer [130]. The knockdown of NEAT1 can efficiently down-regulate the expression of miR-34a and up-regulate the expression of HMGB1, thus inhibiting the expression of autophagy-related proteins Bcl-2 and ULK1 and decreasing the LC3II/I ratio, thus increasing the sensitivity of CRC cells to 5-Fu [131]. Besides, NEAT1 can also be able to enhance the expression of downstream autophagy-related protein ATG3 through exerting a sponge effect on miR-204 and thus stimulating autophagy in hepatocellular carcinoma (HCC) cells and enhancing sorafenib resistance of HCC cells [132].

### LncRNA MALAT1

MALAT1, which is an lncRNA, has been found to be overexpressed in a variety of cancers, such as lung, breast, pancreatic, hepatic, colorectal, gastric, uterine, cervical, and prostate cancers [133, 134]. MALAT1 also possesses the ability to act as an independent survival biomarker for prognosis in the neoplastic cells. Recent research has revealed a correlation between MALAT1 expression and the fate of OS cells, wherein MALAT1 suppression led to the retardation of tumor growth in an OS xenograft model, thereby underlining its oncogenic function and the therapeutic value as a target in the treatment of OS [135]. Furthermore, MALAT1 has been found to enhance OS cell growth and metastasis, most likely by stimulating the phosphoinositide 3-kinase/protein kinase B (PI3K/AKT) signaling pathway. Although such evidence has unraveled a specific correlation between MALAT1 and OS, the individual contribution of MALAT1 to OS tumorigenesis and the underlying mechanism require further elucidation [136].

#### MALAT1 expression and regulation

The MALAT1 gene transcript or NEAT2 (nuclear-enriched abundant transcript 2) contains over 8000 nucleotides, according to the description by Ji et al. [137]. While initial reports had provided evidence for its involvement in metastatic progression in the context of early-stage NSCLC patients, subsequent studies discovered that MALAT1 is extremely widespread and extremely conserved among 33 mammalian species. Subsequently, it was found that MALAT1 is a very highly abundant nuclear transcript localized in nuclear speckles, which are nuclear subcompartments dedicated to storage and/or sites of pre-mRNA splicing; later, computational as well as biochemical investigations verified the co-localization of MALAT1 with serine- and arginine-rich (SR) splicing regulators as well as spliceosome proteins [137].

One of the significant MALAT1 interactors is the TAR DNA-binding protein TDP43, which is localized mainly in the mouse brain. TDP43 is a nucleus-localized protein involved in transcriptional regulation, alternative splicing, and RNA stabilization. In human brains of patients who have been clinically diagnosed with fronto-temporal lobar degeneration with TDP-43 inclusions (FTLD-TDP), the interaction of MALAT1 with TDP-43 was elevated, thereby indicating a potential correlation of MALAT1 with neurodegenerative diseases [138]. MALAT1 is strongly controlled at the transcriptional level in cancerous cells. Numerous transcription factors have been found to function as modulators of MALAT1 transcription, either in a positive role, i.e., SP1, SP3, β-catenin, HIF1α, and HIF2α, c-MYC, Yes-associated protein 1 (YAP1), and NRF1, or in a suppressive role, as demonstrated by p53 and SOX17 [139]. Moreover, hormonal medications, including oxytocin, and growth factors, including TGF-β, are capable of transcriptionally inducing MALAT1, thereby promoting tumor progression. The Drosha-DGCR8 complex, the pivotal component of the miRNA machinery apparatus, has been reported to interact with the 5′ end of MALAT1, which regulates its stability [140]. The role of miRNAs in MALAT1 regulatory mechanism was also confirmed by the research conducted by Leucci et al., who demonstrated that Ago2 depletion resulted in MALAT1 stabilization and, accordingly, indicated that miRNAs are a key player in MALAT1 modulation; notably, the discovery of miR-9 as a MALAT1 degradation target in the nucleus through direct interaction with two distinct miRNA-binding sites on the MALAT1 sequence [141]. In addition, Ago2-mediated post-transcriptional regulation of MALAT1 by miR-101 and miR-217 effectively suppressed the proliferation, migration, and invasion of Esophageal Squamous Cell Carcinoma (ESCC) cells, indicating the tumor-suppressing role of these miRNAs on MALAT1 [142].

A large-scale parallel small RNA sequencing analysis has surprisingly found a range of MALAT1-derived miRNA-like molecules whose biological function remains to be identified. Epigenetic histone marks may also have a regulatory influence on MALAT1 expression. For instance, the Jumonji C-domain-containing protein JMJD1A, previously known to be a H3K9 histone demethylase, is shown to interact with the MALAT1 promoter and demethylate histone H3 at lysine 9 (H3K9), which consequently leads to MALAT1 upregulation [143]. KMD3A, another H3K9 demethylating enzyme, was shown to increase MALAT1 expression in MM cells and hence favor the formation of hypoxic niches supportive of tumor growth. Conversely, lysine-specific demethylase 5B (KDM5B) was shown to reduce MALAT1 expression through the induction of miR-448, which directly targets MALAT1 [144].

### LncRNA–miR-124 networks in cancers

MiRNAs help control many cell functions and are found in similar amounts in different cell types. Multiple roles for miRNAs in carcinogenesis have been elucidated. MiRNAs can be oncogenic, tumor-suppressive, or exhibit dual functions, contingent upon the cancer’s localization [145]. Among these, miR-124 has been identified as a well-preserved tumor suppressor miRNA linked to various cancers [146]. Three genes are responsible for encoding this miRNA: MIR-124-1 (8p23.1), MIR-124-2 (8q12.3), and MIR-124-3 (20q13.33). Expression of the genes that encode miR-124 in different cancer types is regulated via methylation [147–149]. MiR-124 affects many cancer-related genes, such as CBL, PDCD6, ROCK1, SNAI1/2, TWIST1/2, STAT3, and EZH2, and helps control cell growth, development, and specialization; if it is disrupted, it can cause normal cells to become cancerous, which is marked by stopping the cell cycle, changing cell types, spreading to other areas, and becoming resistant to chemotherapy. This miRNA is considered a potential biomarker for the advancement of innovative therapeutic strategies for tumor management [146, 150, 151]. Over the last ten years, it has been shown that cancer-causing lncRNAs and some circular RNAs form networks by competing with other RNAs, following the ncRNA/mir-124/mRNA model. The competitive interactions between mRNA and ncRNA (like lncRNA and circRNA) with miR-124 only happen if there are specific binding sites for this miRNA in the sequences of mRNA and regulatory ncRNAs. Furthermore, oncogenic lncRNA and circRNA impede the function of the suppressor miR-124, thereby preventing it from efficiently inhibiting the translation of its oncogenic protein targets [151].

**Table 1:** Autophagy-related LncRNAs and their signaling pathways in osteosarcoma

LncRNA	Expression level	Role	Sponged miRNA	Target protein/signaling pathway	Effect	Year	Ref.
FGD5-AS1	Upregulated	Oncogene	miR-154-5p	WNT5A	Induces autophagy, proliferation, migration, and chemoresistance of OS cells	2022	[73]
OIP5-AS1	Upregulated	Oncogene	miR-153	ATG5	Promotes autophagy, proliferation, invasion, and angiogenesis; suppresses apoptosis; reduces 5-year survival	2021	[105]
MEG3	Downregulated	Tumor-suppressive	miR-21-5p	p53	Suppresses autophagy, proliferation, and invasion; induces chemosensitivity and anti-tumor immunity	2021	[70]
SNHG15	Upregulated	Oncogene	miR-381-3p	GFRA1	Induces proliferation, autophagy, and chemoresistance	2020	[94]
SNHG15	Upregulated	Oncogene	miR-141	ATG5, LC3-II, p62	Promotes autophagy, proliferation, and migration; suppresses apoptosis; linked to adverse prognosis	2017	[93]
SNHG6	Upregulated	Oncogene	miR-26a-5p	ULK1	Enhances autophagy, proliferation, and invasion; inhibits apoptosis	2019	[116]
LINC00313	Upregulated	Oncogene	miR-342-3p	FOSL2	Promotes proliferation and metastasis; suppresses autophagy and apoptosis; reduces overall survival	2020	[123]
LINC01410	Upregulated	Oncogene	miR-3128	Ki-67 / Cyclin D	Promotes malignant phenotypes in vitro	2020	[120]
Sox2OT-V7	Downregulated (by EGCG treatment)^*^	Tumor-suppressive	NR	Notch3 / DLL3 /	Reduces doxorubicin-induced autophagy, stemness, and chemoresistance; increases OS cell sensitivity to doxorubicin	2018	[85]
Sox2OT-V7	Upregulated	Oncogene	miR-142 / miR-22	ULK1 / ATG4A / ATG5	Induces doxorubicin-induced autophagy and chemoresistance in OS cells	2020	[87]
XIST	Upregulated	Oncogene	miR-375-3p	AKT / mTOR	Promotes autophagy and cell proliferation; inhibits apoptosis	2019	[111]

*NR* Not reported

* EGCG is an exogenous treatment condition that downregulates Sox2OT-V7 expression levels refer to untreated OS cells

## CircRNA: modulators of autophagy in osteosarcoma

CircRNA-mediated autophagy is necessary for maintaining the cellular features of tumor growth, such as proliferation, invasion, and metastasis. It renders cancer cells resistant to chemotherapy and is associated with cancer escape mechanisms. The role that circRNA-mediated autophagy plays in the development of the tumor. Depending on the kind of tumor, circRNA-mediated autophagy sometimes suppresses or promotes cancer growth [152]. In this section, we focus on circRNAs that are involved in autophagy regulation in OS. We emphasize the molecular basis and signaling cascades by which circRNAs influence key downstream mediators of autophagy. The summarized information, including their regulatory axes and functional outcomes, is presented in Table 2.

### CircABCC1

CircABCC1 is a circular RNA derived from the ABCC1 gene, and functions as a miRNA sponge for miRNAs like miR-591. It exerts oncogenic effects, promoting cancer initiation and progression [153]. Wang et al. explored the function of circABCC1 in OS progression and used qRT-PCR, luciferase reporter assays, and functional cell experiments for this aim. They found that circABCC1 is upregulated in OS and promotes tumor growth. Nonetheless, there are a few methodological points to consider. The study utilized a small sample size (37 paired tissue samples) and relied primarily on two cell lines (U2OS and HOS), which limits the applicability of findings to the heterogeneous OS patient population. Moreover, while there was tumor growth inhibition in the xenograft model, the immunocompromised murine model cannot fully replicate the multifaceted tumor microenvironment and immune interactions present in the human disease. MiR-591 is a known target of circABCC1; it has also been directly targeted and inhibited by circABCC1 in OS cell lines [154]. In addition to this direct sponging mechanism, the circABCC1/miR-591/HDAC4 axis may function within more complex regulatory networks, as suggested by evidence for potential crosstalk with the PI3K/AKT and Wnt/β-catenin pathways, which are each commonly dysregulated in OS. There may also be feedback loops whereby HDAC4 may directly modulate the transcription of genes associated with circRNA biogenesis or miRNA processing through its epigenetic regulatory functions. Taken together, a self-reinforcing oncogenic circuit is likely recruited here, but remains incompletely characterized. MiR-591 downregulation leads to increased expression of HDAC4 [154]. The HDAC4 is a member of the histone deacetylases (HDACs) family and can act as both a tumor suppressor and an oncogene in different types of cancer. The context sensitivity in this duality emphasizes the complexity of HDAC4 signaling while also implying potential contributions from additional cellular factors, post-translational modifications, and interactions with other epigenetic regulators, and consequences axiomatic to this notion, that were not addressed in this study, to its role in regulating either side of the autophagy process. In scientific terms, for potential clinical translation, while there may be considerable hope and promise in the potential usage of circABCC1 as a therapeutic target, significant real-world and practical issues will still need to be addressed. Although circRNAs are thought to be stable due to their circular structure, this same circular structure makes it difficult to design targeting therapies. Moreover, efficient delivery systems that target solid tumors by penetrating physiological barriers and producing meaningful intratumoral concentrations are lacking. Current methodologies using either antisense oligonucleotides or small interfering RNAs present potential limitations that include off-target effects, suboptimal tissue penetration, and immunogenicity. It may also be necessary to conduct a full battery of toxicity testing in all normal tissues, given that circABCC1 is highly expressed in normal tissues. The development of specific HDAC4 inhibitors as downstream targets is still an avenue worth exploring for potential use in the clinic through future studies, though the identification and desired specificity associated with HDAC4 targeting while limiting the effect to other family members would prove a challenge in itself. Previous studies demonstrated the regulatory role of HDAC4 in autophagy through epigenetic and non-epigenetic mechanisms [155]. In OS, HDAC4 exerts oncogenic effects, and its expression is upregulated due to the inhibitory effects of circABCC1 on miR-591. Collectively, Wang et al. showed silencing circABCC1 suppressed cell proliferation, migration, and invasion. The results suggest that circABCC1 acts as a ceRNA contributing to OS malignancy. Targeting the circABCC1/miR-591/HDAC4 pathway may represent a promising therapeutic approach [154].

### CircMRPS35

CircMRPS35 is a tumor-suppressive circRNA with a particularly significant effect in suppressing chemoresistance. Previous studies have shown that circMRPS35 inhibits gastric cancer and OS progression [156, 157]. Unlike many circRNAs that function via miRNA sponging, circMRPS35 exerts its tumor-suppressive roles through interaction with chromatin modifiers. Jiang et al. investigated the role of circMRPS35 in OS progression and autophagy regulation. They revealed that downregulation of circMRPS35 reduced KAT6B function, a histone acetyltransferase known for activating transcription through chromatin remodeling, which led to reduced transcription of FOXO3 [158]. FOXO3 is a transcription factor that plays a dual role in cancer, notably promoting autophagy by activating genes such as LC3 and Beclin1 [159, 160]. Reduced FOXO3 expression, driven by circMRPS35/KAT6B interaction, led to lower autophagy and increased tumor cell proliferation. In OS, this autophagy appears to inhibit tumor survival rather than enhance it. The depletion of circMRPS35 repressed the FOXO3-mediated autophagy and malignant phenotypes of OS cells. Together, the study identifies the circMRPS35/KAT6B/FOXO3 axis as a novel epigenetic regulatory pathway contributing to suppressing OS progression and highlights it as a potential therapeutic target [158]. In line with this, Jiang et al. also reported that circMRPS35 is downregulated in OS, and inducing its overexpression can inhibit OS progression by sponging miR-105-5p and restoring FOXO1 expression. FOXO1 is another forkhead transcription factor with known tumor-suppressive and autophagy-regulating roles. This complementary work further supports the dual functional significance of circMRPS35 in OS via distinct but related FOXO-mediated pathways [157].

### CircKMT2D

CircKMT2D has recently emerged as a key player in oxidative stress-related tumor regulation. In a recent study, Zhang et al. investigated how circKMT2D responds to hydrogen peroxide (H₂O₂)-induced oxidative stress in OS cells, and how it modulates autophagy. Using qRT-PCR, Western blotting, dual-luciferase assays, and gain/loss-of-function experiments, they showed that circKMT2D is highly expressed in OS cell lines and its expression is significantly reduced upon H₂O₂ treatment, leading to suppressed OS cell viability. CircKMT2D was found to function as a miR-210 sponge [161]. MiR-210 plays a dual role in regulating autophagy, and is known to inhibit autophagy-related genes under stress and hypoxia conditions [162]. Zhang et al. found that downregulation of circKMT2D under oxidative stress led to miR-210 accumulation, which in turn repressed key autophagy markers like Beclin1 and LC3-II, contributing to impaired autophagic flux. Importantly, this regulatory axis has a context-dependent bidirectionality, as mir-210 inhibited autophagy when induced oxidative stress occurred via H2O2 addition, while circKMT2D’s sponging activity appeared to function as a feedback loop, in which circKMT2D stability increased without autophagy, suggesting some type of homeostatic mechanism that promotes tumor cell survival. The interplay between the circKMT2D/miR-210 mechanism discussed earlier with the PI3K/AKT/mTOR pathway, the master regulator of the autophagy program, is still incomplete. However, the existing literature on circKMT2D, miR-210, and autophagy suggests potential crosstalk, as inhibiting mTOR signaling may affect circKMT2D, as well. In addition, epigenetic modifications such as H3K27me3 enrichment at the KMT2D locus may also affect circKMT2D biogenesis, suggesting another layer of complexity and bio-regulatory scope that should be investigated. Restoring circKMT2D expression reversed these effects, promoting autophagy and supporting tumor cell survival even under oxidative pressure. However, these findings were based solely on in vitro studies of MG63 and U2OS cells, without confirming their relevance in patient-derived xenograft models or clinical samples, hindering their translational relevance. The relatively small sample size of the study (*n* = 3 replicates) and transient transfection methods likely do not mimic the robust and stable regulation of circKMT2D observed in the tumor microenvironment over an extended period of time. Furthermore, mechanistic interpretation is further compromised by a lack of rescue experiments, besides only using the neutrophil autophagy inhibitor 3-MA. These findings suggest that circKMT2D plays a protective oncogenic role in OS by modulating the miR-210/autophagy axis in response to oxidative stress. Targeting this circRNA could enhance OS sensitivity to oxidative damage and represent a potential therapeutic target. However, several important hurdles must be overcome before any clinical adoption. First, while the stability of circRNAs is productive for developing biomarkers, the circular nature of the RNA makes them less effective for delivery in vivo, since currently available delivery methods, such as viral vectors or lipid nanoparticles, lack specificity for targeting bone tissue and can induce an immunogenic response. Second, the practical assumption that circKMT2D can be detected in peripheral blood or bone marrow aspirates in a non-invasive diagnostic setting must be addressed in large multi-site cohort studies alongside a standardized process of RNA extraction and quantification. Finally, safety remains a top priority: should we inhibit circKMT2D in the serum or in the bone, we will be disrupting autophagy along with every osteoblast or other rapidly dividing tissue that could be affected, and we will need to implement robust toxicity studies in immunocompetent animal studies as well as off-target effects by transcriptome-wide binding site analysis [161].

**Table 2:** Autophagy-related circrnas and their signaling pathways in OS

CircRNA	Expression level	Role	Sponged miRNA	Target protein/signaling pathway	Effect	Year	Ref.
CircABCC1	Upregulated	Oncogene	miR-591	HDAC4	Increasing viability, proliferation, migration, and invasion of OS cells Decreasing apoptosis and autophagy; promoting tumor growth in vivo	2023	[154]
CircMRPS35	Upregulated	Tumor-suppressive	NR	KAT6B/FOXO3	Suppressing OS cell proliferation, migration, and invasion via altering epigenetic regulation	2020	[158]
CircKMT2D	Downregulated	Oncogene	miR-210	Beclin1, p62	Increasing viability and invasion in OS cells Decreasing apoptosis and autophagy upon circKMT2D knockdown	2020	[161]

*NR* Not reported

## LncRNAs and circrnas as promising therapeutic targets in osteosarcoma

The current standard treatment of OS consists of surgery to remove the neoplasm, supplemented with adjuvant chemotherapy. While targeted pharmacological agents are being developed to treat OS, none have so far received approval from the regulatory agencies. Chemoresistance is the frequent occurrence during the therapy of OS. Hence, the prognosis of OS patients has not been significantly enhanced over a long period, which is attributed, at least in part, to the absence of appropriate therapeutic targets and the prevalence of chemoresistance. Hence, the identification of novel therapeutic targets is necessary to enhance the prognosis of patients with OS.

LncRNAs and circRNAs have become appealing contenders for such purposes, with their cell-, tissue-, and disease-specific expression patterns, and pre-clinical models demonstrating encouraging results in the context of a wide range of disorders, including cancers [163]. Furthermore, targeting lncRNAs and circRNAs provides various advantages over conventional protein-targeting strategies because the base-pairing principles are comparatively simpler than designing specific inhibitors for targeting protein interactions. LncRNAs can be targeted using various approaches, such as small interfering RNA (siRNA), antisense oligonucleotides (ASOs), ribozymes, aptamers, and miRNAs, all of which have been tested for their potential to target cancer-related genes [164]. For example, Arun et al. treated mice with a 16-mer gapmer to MALAT1 modified with alternating constrained nucleotides and phosphorothioate linkages via subcutaneous injection before the spontaneous formation of mammary tumors. Treatment resulted in a 50% reduction in metastatic burden and a highly differentiated cystic/ductular histologic phenotype reminiscent of that observed in MALAT1−/− genomic knockout mice [165]. In the same way, PDX mouse models of patient-derived xenografts indicated that treatment with lipid nanoparticle (LNP)-formatted anti-HOXB-AS3 gapmers strongly downregulated HOXB-AS3 expression in human CD45-selected acute myeloid leukemia (AML) blasts obtained from the bone marrow of mice being treated. This treatment significantly extended the survival of the treated mice over the control group, as shown by the median survival of scramble-treated mice being just 63 days, while the survival of the HOXB-AS3 knockdown group was still unmet even after 100 days of treatment. LncRNAs associated with drug resistance, such as LUCAT1, ANRIL, and Sox2OT-V7, may have the potential to be therapeutic targets to reverse chemotherapy resistance in the future. Additionally, lncRNAs with dysregulated expression in OS, such as LINC00588, GAPLINC, HOTAIR, and DNACR, that regulate the metastatic potential of OS can be investigated for therapeutic targeting [166]. For instance, lncRNA LINC00588 is variably expressed in osseous cancer when compared to metastatic lung carcinoma and may function as a ceRNA in modulating the expression of TP53. Thus, the LINC00588/miRNA-1972/TP53 axis may be an effective therapeutic target in OS patients [167].

## Critical appraisal and future directions

Although numerous research studies have sought to elucidate the role of lncRNAs and circRNAs in autophagy regulation in OS, most of the existing data are from in vitro or small-scale investigations, whose translational applicability is limited. The majority of reports are based on single cell lines and lack confirmation using clinic samples, thereby limiting an estimation of the reproducibility and biological relevance of reported outcomes. Moreover, most studies report molecular interactions (e.g., lncRNA–miRNA–mRNA axes) without showing causal mechanisms or upstream regulators of the networks. A parallel challenge is inconsistency across studies. While some studies indicate that induction of autophagy enhances survival of OS cells and chemoresistance, others show that overactivation of autophagy induces apoptosis and growth inhibition. These differences may result from heterogeneity in experimental protocols, treatment conditions, or cell-type–dependent regulatory mechanisms. In addition, less has been studied about how ncRNA-induced autophagy is associated with other pathways such as PI3K/AKT/mTOR, Wnt/β-catenin, and p53 in the microenvironment of a tumor. The association of autophagy with immune modulation, oxidative stress, or metabolic reprogramming in OS has yet to be elucidated. Future studies should prioritize in vivo validation, employ high-throughput multi-omics technology, and employ patient-derived models to validate ncRNA–autophagy networks within clinically relevant contexts. The integration of bioinformatics prediction with experimental verification can uncover master regulators that are amenable to be employed as diagnostic biomarkers or therapeutic targets. A systematic examination of conflicting evidence will also shed light into whether autophagy is a predominantly tumor-suppressive or tumor-supportive process under different biological conditions.

## Conclusion

The results demonstrate the potential use of lncRNAs and circRNAs and their corresponding regulatory axis for developing new diagnostic, prognostic, and therapeutic strategies in OS. LncRNAs or their associated proteins may overcome drug resistance, inhibit tumor progression, and enhance patient outcomes. LncRNAs can alter the activity of cancer-related signaling pathways, including the PI3K/AKT, Hippo, Notch, TGF-β, Wnt/β-catenin and JAK/STAT pathways. The PI3K/AKT signaling pathway was the most reported pathway impacted by lncRNAs within OS. Further study is essential to understand their complex biological underpinnings, validate their clinical efficacy, and develop viable treatment methods for OS. Because of the potential robust interplays of lncRNAs with miRNAs, it is not feasible to evaluate the contribution of lncRNAs in OS without a complete characterization of the miRNA signature. This approach may lead to the recognition of lncRNA-miRNA pairs with potential for biomarkers or therapeutic applications. SNHG15, FOXC2-AS1, and OIP5-AS1 are examples of lncRNAs whose levels of transcripts have been shown to predict chemotherapy response. Given the importance of these agents in prolongation of survival for OS patients, directed therapeutic interventions against these transcripts are potential candidates for enhancing cytotoxicity against OS. Unfortunately, the majority of these research studies have been completed in cell lines, and we cannot expect to directly translate these studies to clinical applications. An unanswered important question is how to target pathogenic lncRNAs in a tissue-selective manner. It is plausible to utilize tissue-specific vectors that will bind to specific surface markers on OS cells or deliver lncRNA-targeting siRNAs locally, and the effectiveness of these approaches could be assessed in animal models and patients. Other therapeutic options should be explored to target lncRNAs, including short hairpin RNAs, which may possess transient or stable gene silencing activities, ASOs of various designs, including locked nucleic acid GapmeRs, antagonists to natural antisense RNAs, and mixmers, along with deoxyribozymes as enzymatic DNA molecules, and genome editing methods such as the CRISPR/Cas9 system. The feasibility of using lncRNA-targeted therapeutics would need to be examined in animal models before considering clinical feasibility.

circRNAs have emerged as key regulators of autophagy and oncogenesis in OS that operate through complex ceRNA networks regulating proliferation, metastasis, and chemoresistance. In general, the studies compiled in this review illustrate how specific circRNAs can induce or repress autophagy by targeting autophagy-stimulating key pathways such as PI3K/AKT/mTOR, Notch, and p53 signaling. These findings shed new insights into the molecular mechanisms of OS and reveal promising candidates for diagnosis and treatment.

Future opportunities encompass the validation of lncRNAs and circRNAs as effective biomarkers for early diagnosis, prognosis, and response prediction to treatment in OS. The therapeutic use of these unique ncRNA-targeting therapies remains restrained by issues of delivery, stability, and off-target toxicity; therefore, effective nanocarrier or exosome-delivery systems will be required. Furthermore, the integration of multi-omics data and the principles of systems biology could improve the identification of clinically useful ncRNA signatures. Of particular significance is that clinical trials using ncRNA mimics or inhibitors are just beginning, and their extension to patients with OS will help to span the bench-to-bedside gap. Overall, increased mechanistic understanding of lncRNAs and circRNAs-controlled autophagy, combined with technological innovation in RNA therapeutics, holds considerable promise to ameliorate OS therapy and patient prognosis.

LncRNAs and circRNAs play central roles in the regulation of autophagy during OS progression. In addition to their involvement in autophagy, they influence cell proliferation, invasion, and chemotherapeutic response in OS tissues and cell models. Dysregulated expression of these ncRNAs highlights their potential as biomarkers and therapeutic targets. A deeper understanding of their spatial and temporal dynamics in autophagy regulation facilitates the development of more effective and targeted therapeutic strategies for OS management.

## Data Availability

No datasets were generated or analysed during the current study.
